# Effects of Protective Resin Coating on the Surface Roughness and Color Stability of Resin-Based Restorative Materials

**DOI:** 10.1155/2014/832947

**Published:** 2014-08-05

**Authors:** Bora Bagis, Tamer Tüzüner, Sedanur Turgut, Fatih Mehmet Korkmaz, Özgül Baygın, Yıldırım Hakan Bağış

**Affiliations:** ^1^Department of Prosthodontics, Faculty of Dentistry, Izmir Katip Celebi University, Izmir, Turkey; ^2^Department of Pediatric Dentistry, Faculty of Dentistry, Karadeniz Technical University, Trabzon, Turkey; ^3^Department of Prosthodontics, Faculty of Dentistry, Karadeniz Technical University, Trabzon, Turkey; ^4^Department of Restorative Dentistry, Faculty of Dentistry, Ankara University, Ankara, Turkey

## Abstract

The aim of this study was to evaluate the effects of nanofilled protective resin coating (RC) on the surface roughness (Ra) and color stability (Δ*E*) of resin-based restorative materials (RM) (compomer (C), nanofilled composite (NF), and microhybrid composite (MH)) after being submitted to the ultraviolet aging (UV) method. Thirty-six specimens were prepared (*n* = 6 for each group). The Ra and (Δ*E*) values and SEM images were obtained before and after UV. Significant interactions were found among the RM-RC-UV procedures for Ra (*P* < 0.001). After the specimens were submitted to UV, the Ra values were significantly increased, regardless of the RC procedure (with RC; *P* < 0.01 for all, without RC; C (*P* < 0.01), NF (*P* < 0.001), and MH (*P* < 0.001)) for each RM. Significant interactions were found between the RM-RC (*P* < 0.001) procedures for the Δ*E* values. The Δ*E* values were increased in each group after applying the RC procedures (*P* < 0.001). Protective RC usage for RM could result in material-related differences in Ra and Δ*E* as with used UV method.

## 1. Introduction

Tooth-colored composite materials have been widely used for aesthetic purposes [1–5]. Compomers, defined as “polyacid-modified resin composites,” were introduced in the dental literature in the early 1990s and have commonly been used for primary and permanent tooth restorations [6, 7]. Composites and compomers must have smooth surfaces to inhibit plaque accumulation [7–11].

In clinical situations, the longevity of restorations is commonly related to acceptable finishing and polishing properties, which provide smooth surfaces [4, 12, 13]. Surface coating procedures have been reported as beneficial methods for decreasing the rougher properties of dental resin-based restorative materials (RM) [8, 14–17]. Furthermore, in such cases, surface coatings have not been able to fill whole surface irregularities [18, 19].

Higher surface roughness (Ra) values (>0.2 *μ*m) have been reported as a risk factor for extensive plaque accumulation on dental materials and as the main contributor to the multifactorial discoloration of resin restorations [4, 8, 12, 20, 21], which is strongly correlated with the inorganic fillers in RM [20, 22–24]. The surface degradation and color stability characteristics of RM, without [12, 20] or with [8, 25] surface coatings, can be affected by several factors, including filler type, size [26], or exposure to colorant [2, 4, 8]. Moreover, the surface resistance of RM, with decreased filler particle sizes (<1 *μ*m), is not able to advance using surface coating materials [4, 18]. Additionally, the coherence properties of surface coatings, which can alter the penetration into the restoration surface, have been evaluated in the literature [27–30]. The long-term durability of these coatings with RM after implementing various aging procedures has shown that coatings can debond over time, which has been noted to be important by many researchers [8, 12].

Ultraviolet aging (UV) is a method that simulates clinical conditions, allowing the color differences of materials over time to be determined. As materials are exposed to a range of conditions, including UV light, temperature changes, and continuous humidity, artificially accelerated aging simulates clinical parameters as closely as possible [31]. This technique has been used to investigate the Ra and discoloration of dental materials [32–34]. Nevertheless, the durability of these surface coatings on RM and their possible long-term effects remain unknown.

The aim of this study was to evaluate the effects of nanofilled protective resin coating (RC) on the surface roughness (Ra) and color stability (Δ*E*) of RM (compomer (C), nanofilled composite (NF), and microhybrid composite (MH)) after being submitted to UV tests. The null hypothesis of this study was that the RC procedure would not change the surface roughness or color values of the RM after UV.

## 2. Materials and Methods

### 2.1. Specimen Preparation

Resin-based restorative materials, one compomer (C) and two composite resins (NF and MH), with or without the resin coating (RC), were used in this study with the shade of A3 (Table 1). A total of 36 disk-shaped specimens (10 mm in diameter and 2 mm in height) were prepared, covered with clear strips and light cured perpendicularly (Elipar FreeLight 2, 3 M ESPE, St. Paul, MN, USA, for 20 s) in plastic molds for both the Ra and Δ*E* tests (*n* = 6 for each group). After polymerization was completed, the specimens were divided into two groups, and half were treated with the RC by using microtip applicator with the same above light curing device for 20 s. Then, the specimens were stored at 37°C and 100% relative humidity for 24 hours to ensure complete polymerization. One specimen, before and after the UV testing from each group, was stored for scanning electron microscopy (SEM) analysis. The tested groups are shown in Table 1.

### 2.2. Ultraviolet Aging (UV)

The specimens were subjected to UV using an Atlas UV 2000 testing machine (Material Testing Technology LLC, Chicago, IL, USA). Aluminum plates were prepared in accordance with the sample sizes, and the specimens were inserted into the molds of the plates and subjected to aging tests. All of the specimens were exposed to ultraviolet light and water spray for 300 hours in the testing machine. The glazed surface of each specimen was continuously exposed to the light source. The back panel temperature ranged between 38°C (dark) and 70°C (light), and the relative humidity was 95% (dark) or 50% (light). The dry bulb temperature was 38°C in the dark stage and 47°C in the light stage. The testing cycle consisted of 40 minutes of light only, 20 minutes of light with a front water spray, 60 minutes of light only, and 60 minutes in the dark with a back water spray. The total exposure energy was 150 kJ/m^2^.

### 2.3. Surface Roughness (Ra)

The average surface roughness of the specimens was measured with a surface profilometer (MarSurf PS1; Mahr, GmbH, Göttingen, Germany). To measure the roughness profile value, the diamond stylus (5 *μ*m tip radius) was moved across the surface under a constant load of 3.9 mN. The instruments were calibrated by using a standard reference specimen and then set to travel at a speed of 0.100 mm/s with a range of 600 *μ*m during testing. Surface roughness was measured 5 times for each specimen in the central part; the average value was obtained and defined as the Ra.

### 2.4. Color Stability (Δ*E*) Evaluation

The color measurements were obtained with a colorimeter (ShadeEye NCC, Shofu, Japan) in a viewing booth, under D65 standard illumination on a white background, and these measurements were based on the ISO standards (ISO 7491). Before the experimental measurements, the colorimeter was calibrated according to the manufacturer's instructions, and it was positioned in the middle of each sample. The L*a*b* color notation of each specimen was measured consecutively three times, and the average of the three readings was calculated to yield the initial color of the specimen. The Commission Internationale de l'Eclairage (CIE) system was used to evaluate the Δ*E* (i.e., the degree of perceptible color change) based on three coordinates: L*, a*, and b*.* L* (lightness or brightness value) corresponds to the L* of the CIE Lab* system and represents the lightness/darkness of a color; a* is a measurement of redness (positive) or greenness (negative); and b* is a measurement of yellowness (positive) or blueness (negative). The CIE color difference is calculated with the following equation: Δ*E* = [(ΔL*)^2^+(Δa*)^2^+(Δb*)^2^]^1/2^.

### 2.5. Scanning Electron Microscopy (SEM)

Samples were randomly selected from each group (before and after UV with RC) and gold-coated with an ion coating unit (Polaron SC 500 Sputter Coater; Quorum Technologies, Ashford, UK). Those samples were then evaluated and photographed under a SEM (EVO L10; Carl Zeiss, Oberkochen, Germany) to determine the surface alterations.

### 2.6. Statistical Analysis

Statistical evaluations were performed with statistical software (SPSS v15.0 for Windows; SPSS Inc., Chicago, IL, USA). Three-way ANOVA and Fisher's LSD test were used for analyzing the Ra values and two-way ANOVA and Fisher's LSD were used for comparing the Δ*E* values at a confidence interval of 95%.

## 3. Results

### 3.1. Surface Roughness (Ra)

Three-way ANOVA revealed significant interactions between the RM-RC (*P* < 0.001), RM-UV (*P* < 0.001), RC-UV (*P* < 0.001), and RM-RC-UV (*P* = 0.028) for the Ra values (Table 2).

The surface roughness (Ra) values of the groups were shown in Table 3. The MH samples exhibited significantly lower values in the without RC/before UV procedures than the C and NF samples (*P* = 0.003). In the without RC/after UV procedures, the MH samples showed significantly higher values than the NF (*P* = 0.003) and C (*P* = 0.001) samples. In the with RC/before UV procedures, no significant differences were found among the groups (*P* > 0.05). In the with RC/after UV procedures, the MH samples showed higher values than the NF (*P* = 0.007) and C (*P* = 0.003) samples.

The without RC procedures after UV conditions revealed that the Ra values were significantly higher in the C (*P* = 0.007), NF (*P* < 0.001) and MH groups (*P* < 0.001) compared to the before UV conditions. The with RC procedures revealed that Ra values were significantly higher for after UV conditions in the C (*P* = 0.005), NF (*P* = 0.006), and MH groups (*P* = 0.007) compared to the before UV conditions. In all of the groups, no significant differences were found between the with and without RC procedures under the before UV conditions (*P* > 0.05). However, significant differences were found between the with and without RC procedures in the after UV conditions for the C (*P* = 0.028), NF (*P* = 0.001), and MH (*P* < 0.001) materials.

### 3.2. Color Stability (Δ*E*)

Two-way ANOVA for Δ*E* (which was calculated from L*a*b* difference between before UV and after UV procedures) revealed significant interactions among the RM-RC procedures (*P* < 0.001) (Table 4).

Δ*E* values of the groups are shown in Table 5. The MH samples had significantly lower values than the NF (*P* < 0.001) and C (*P* < 0.001) samples without the RC procedure. In the with RC procedures, significantly lower values were found in the C group than in the NF (*P* = 0.02) group, and the MH samples also showed significantly lower values than the C (*P* < 0.001) and NF (*P* < 0.001) samples. In all of the tested individual RM, the Δ*E* values were significantly higher in the with RC procedures, than in the without RC procedures (*P* < 0.001).

### 3.3. SEM Evaluations

**Figure 1:**
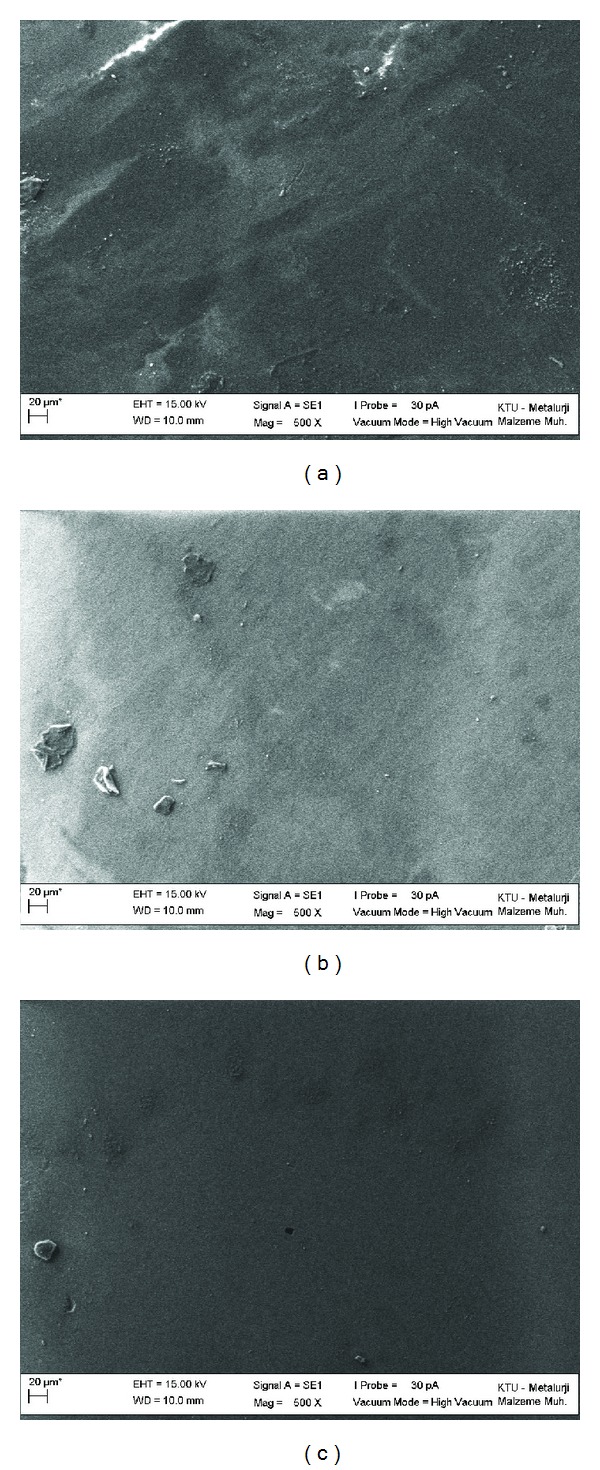
SEM evaluations of the samples with RC before UV. (a) Compomer, (b) nanofilled composite, and (c) microhybrid composite (Mag ×500).

**Figure 2:**
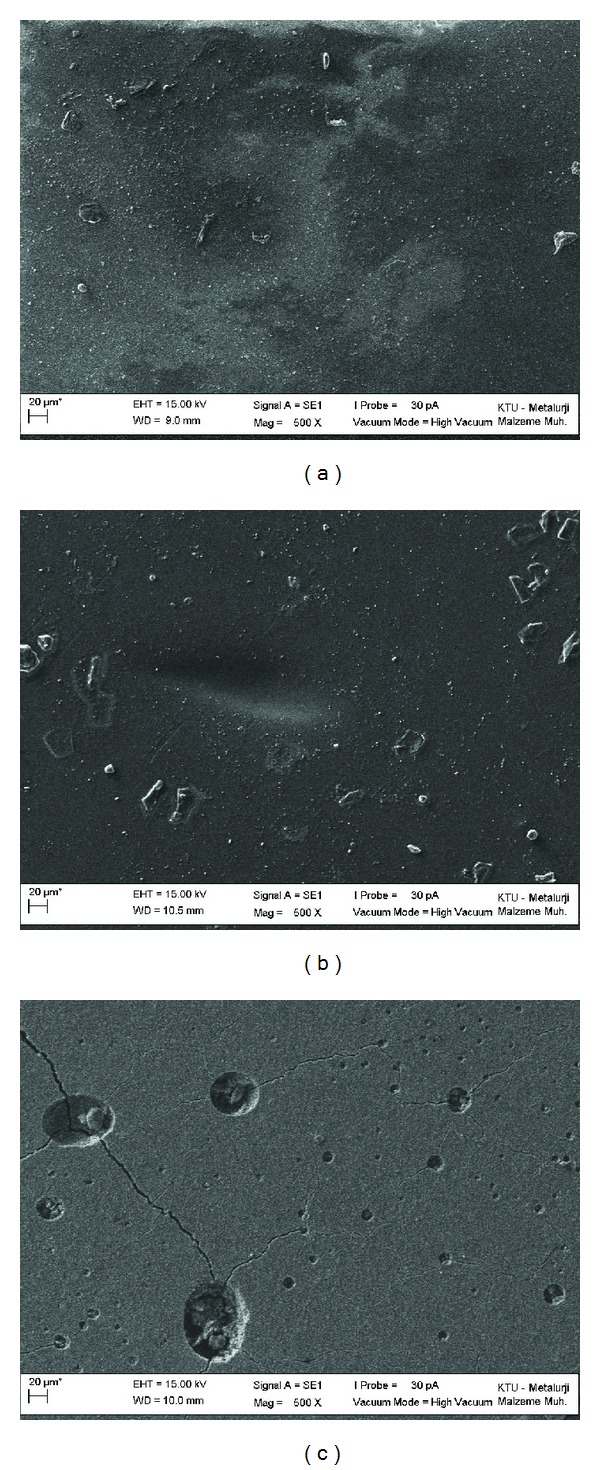
SEM evaluations of the samples with RC after UV. (a) Compomer, (b) nanofilled composite, and (c) microhybrid composite (Mag ×500).

According to the SEM findings, all of the tested materials showed almost smooth surfaces before UV procedure, irrespective of the material property of C (Figure 1(a)), NF (Figure 1(b)), and MH (Figure 1(c)). However, rougher surface irregularities were observed after the UV in C (Figure 2(a)) and NC (Figure 2(b)) of the groups with the RC procedure. The most prominent rough surface irregularities were obtained in the MH composite group with the RC procedure after UV (Figure 2(c)).

## 4. Discussion

The null hypothesis of this study was rejected. The coating procedure resulted in altered Ra and Δ*E* values in all of the tested groups after being submitted to the UV procedure.

The most common method for testing the effects of coating procedures on the surface texture of materials has been reported as using these sealants with previously applied conventional polishing procedures [4, 7, 8, 12, 35, 36]. Thus, less rough surfaces could be obtained without the presence of defects, which resulted from the finishing and polishing procedures. However, such findings revealed that the thin layer of surface coating material might eliminate the surface irregularities or defects of inadequately polished composite restorations [8] and that this procedure also had little effect on previously polished surfaces [18]. In this study, to eliminate the potential beneficial effects of coating procedures on grinded polished surfaces, only a Mylar strip was used. Although this technique has not been commonly used in this type of study, it might be considered a “worst case scenario” for clinical conditions compared to the polished surfaces since the polishing quality depends upon the time-consuming properties of the operators [13].

The manufacturer of the UV machine used in the present study has claimed that 300 h of accelerated weathering (150 kJ/m^2^) is equivalent to 1 year of clinical service [31]; however, clinical validation of this claim is not available. Ultraviolet exposure with temperature and humidity changes might better simulate the oral environment [12, 36–38]. In the present study, the specimens were aged for 150 kJ/m^2^ because RM has been reported to undergo the most significant changes during this initial period [38]. Moreover, the Ra values, which were less than 0.2 *μ*m before the aging procedure, indicated clinically acceptable smooth surfaces for all of the groups, irrespective of the material property or RC procedure, even after the Mylar strip was used (Figures 1(a), 1(b), and 1(c)). In previous reports, different aging procedures were used to measure the Ra values of the RM [4, 8, 12]. However, no clear evidence was found regarding the effects of the coating procedures on the RM, particularly after the materials were submitted to different aging procedures. After being submitted to the UV procedure, all of the tested materials had significantly higher and clinically unacceptable Ra values (>0.2 *μ*m) than their before UV measurements, irrespective of the RC procedure for all tested RM. This feature might be considered a material-dependent factor, which could be related to the aging method that is used in this study.

Before the UV procedure, the surface textures of RM with RC were found similar (Figures 1(a), 1(b), and 1(c)). After the UV, the SEM findings revealed prominent surface irregularities with more debonded and cracked surface features of the RC material in the MH group (Figure 2(c)) compared with the other groups (C and NF) (Figures 2(a) and 2(b)). The debonded feature of the coating procedure obtained in all of the tested materials exhibited material-dependent coherence properties. Because the same manufacturer as the RC material fabricated the MH composite, the surface areas could potentially be attached powerfully while detaching the cracked surface layers and subsequently exposing the rougher subsurface areas after the UV. Although the C group had a similar filler size to the MH material, significantly lower Ra values were obtained after UV. This finding could be related to the glass polyalkenoate composition being identical to that of the glass ionomers. Previous researchers have indicated that the coating procedure could properly seal the porosities and cracks in glass ionomers [16, 17]. With the poorer surface finishing properties of C materials compared with composites [11], the application of a thin layer of coating material would increase the likelihood of fewer rougher surfaces being exhibited after UV. According to this finding, the possible benefits of using C materials to coat teeth, particularly primary teeth [6, 7], should not be overlooked because of their provisional usage in the pediatric population.

Increased Ra is also known to be a major predisposing factor for the extrinsic discoloration of RM [8, 20, 22, 24]. To simulate the oral environmental conditions and to determine the Δ*E* of RM, several* in vitro* methods, including storage in water, dark and dry situations, exposure to UV radiation, and exposure to staining solutions, have been used [8, 33]. Color stability can be obtained visually and by colorimetry or spectrophotometry [20, 21]. Additionally, few studies have stated that coating procedures have negative effects on the Δ*E* of RM with different aging procedures [8]. Nevertheless, the effects of coating and the aging-related discoloration differences among coatings and dental materials remain unknown. For the above-mentioned reasons, the Δ*E* values of the tested materials (with or without RC) were evaluated after being submitted to the UV.

Clinically reasonable color change values were interpreted as being <3.5 [33, 38]. In the present study, all of the groups exhibited Δ*E* values greater than 3.5 after UV, except for the MH group (3.34 Δ*E*), which was considered clinically unacceptable. Color changes in RM induced by UV irradiation have been correlated with chemical alterations in the initiator system, the activators, and the resin itself. The degradation of residual amines and oxidation of residual unreacted carbon-carbon double bonds culminate in the formation of yellowing compounds [36–38]. In addition, the physicochemical properties of the monomers used in a resin matrix can influence resistance to staining [37]. As these materials age, the water sorption characteristics of the resin monomers could contribute to differences in the degree of Δ*E* [33, 38]. In the present study, all of the materials showed significantly increased Δ*E* patterns with the RC procedure compared with their counterparts without RC. The RC procedures can result in prominent cracked and rougher surfaces after UV; the most prominent Δ*E* value was found in the NF group. This finding might have been due to the chemical alterations of these RM after UV.

These findings indicate that every restorative dental material requires its own treatment modality to obtain and maintain surfaces that are as smooth as possible. The protective RC of RM might be a risk factor for Ra and Δ*E*.

## 5. Conclusions

Within the limitations of this study, the following conclusions can be drawn.

1. Protective RC usage for RM might not be an advantage for the materials' Ra and Δ*E* in the long term.
2. The Ra and discoloration values of RM increased after UV.
3. Protective RC usage for RM might result in more discolored and rougher surfaces than without RC.
4. Protective RC usage for RM could result in material-related differences in Ra and Δ*E* in the long term.

## Figures and Tables

**Table 1:** Composition of the materials.

Material	Manufacturer	Composition	Filler size	Lot number	Shade
Compomer	Dyract eXtra (Dentsply DeTrey, Konstanz, Germany)	Urethane dimethacrylate (UDMA), Carboxylic acid modified dimethacrylate (TCB resin), triethyleneglycol dimethacrylate (TEGDMA), trimethacrylate resin, camphorquinone, ethyl-4-dimethylaminobenzoate, butylated hydroxytoluene (BHT), UV stabiliser, strontium-alumino-sodium-fluoro-phosphor-silicate glass, highly dispersed silicon dioxide, strontium fluoride, iron oxide, and titanium dioxide pigments	0.8 *μ*m	11004002055	A3

Nanofilled composite	Nanosit (Nordiska Dental AB, Ängelholm, Sweden)	Silanated barium glass, bisphenol A diglycidylmethacrylate (BisGMA), 1,6-hexanediol dimethacrylate, fumed silica, ethyl 4 dimethylaminobenzoate, camphorquinone, titanium dioxide, Dye (iron oxides), 2,4-dihydroxybenzophenone, and butylated hydroxyl toluene	7 nm	0510	A3

Microhybrid composite	Gradia Direct X (GC Co., Tokyo, Japan)	Urethane dimethacrylate (UDMA), bisphenol A diglycidylmethacrylate (BisGMA), Fluoro-alumino-silicate glass, silica powder, prepolymerized filler, dimethacrylate, and camphorquinone	0.85 *μ*m	1104073	A3

Nanofilled coating	G-Coat Plus (GC Co., Tokyo, Japan)	Methylmethacrylate, multifunctional methacrylate, and camphorquinone	35–40 *μ*m nanofiller particles	0908061	

**Table 2:** Three-way ANOVA table for interactions of Ra values.

Interactions	Sum of squares	df	Mean square	*F*	*P*
RM-RC	0.381	2	0.190	10.824	<0.001
RM-UV	0.622	2	0.311	17.677	<0.001
RC-UV	0.269	1	0.269	15.266	<0.001
RM-RC-UV	0.136	2	0.068	3.867	=0.028

**Table 3:** Surface roughness (Ra) values of the groups (mean ± SD).

Groups (*n* = 5)	Without RC		With RC	
	Before UV	After UV	Before UV	After UV
C	0.19 ± 0.07^A,a,1^	0.29 ± 0.03^A,b,2^	0.17 ± 0.02^A,a,1^	0.37 ± 0.09^A,b,3^
NF	0.19 ± 0.06^A,a,1^	0.30 ± 0.05^A,b,2^	0.18 ± 0.03^A,a,1^	0.46 ± 0.03^A,b,3^
MH	0.07 ± 0.02^B,a,1^	0.41 ± 0.07^B,b,2^	0.19 ± 0.08^A,a,1^	1.06 ± 0.09^B,b,3^

In each column (among the RM), the same superscript capital letters indicate no significant differences with respect to their UV or RC procedures (*P* > 0.05).

In each row (for the individual RM), the same lowercase superscript letters indicate no significant differences (*P* > 0.05), but different letters indicate significant differences before and after UV for the individual RC procedure (*P* < 0.05).

In each row (for individual conditions of the UV procedure; before or after), the same numbers indicate no significant differences (*P* > 0.05), but different numbers indicate significant differences without and with the RC procedure (*P* < 0.05).

**Table 4:** Two-way ANOVA table for interactions of Δ*E* values.

Interactions	Sum of squares	df	Mean square	*F*	*P*
RM	154.655	2	77.328	126.727	<0.001
RC	197.543	1	197.543	323.740	<0.001
RM-RC	28.541	2	14.271	23.387	<0.001

**Table 5:** Δ*E* values of the groups (mean ± SD).

Groups (*n* = 5)	Without RC (after UV)	With RC (after UV)
	Δ*E* values	Δ*E* values
C	6.21 ± 0.90^A,a^	9.28 ± 0.47^A,b^
NF	5.73 ± 1.34^A,a^	12.38 ± 0.44^B,b^
MH	3.34 ± 0.48^B,a^	5.68 ± 0.64^C,b^

In each column (among the RM), the same superscript capital letters indicate no significant differences (*P* > 0.05) with respect to their UV or RC procedures (*P* > 0.05).

In each row (for the individual restorative material), the same lowercase superscript letters indicate no significant differences (*P* > 0.05), but different letters indicate significant differences before and after UV for the individual RC procedure (*P* < 0.05).
